# Completeness and reliability of mortality data in Viet Nam: Implications for the national routine health management information system

**DOI:** 10.1371/journal.pone.0190755

**Published:** 2018-01-25

**Authors:** Tran Thi Hong, Nguyen Phuong Hoa, Sue M. Walker, Peter S. Hill, Chalapati Rao

**Affiliations:** 1 Fundamental Sciences Faculty, Hanoi University of Public Health, Hanoi, Viet Nam; 2 School of Public Health, University of Queensland, Brisbane, Australia; 3 Family Medicine Department, Hanoi Medical University, Hanoi, Viet Nam; 4 School of Public Health and Social Work, Queensland University of Technology, Brisbane, Australia; 5 National Centre for Health Information Research and Training, Queensland University of Technology, Brisbane, Australia; 6 Department of Global Health, Research School of Population Health, Australian National University, Canberra, Australia; University of Washington, UNITED STATES

## Abstract

**Background:**

Mortality statistics form a crucial component of national Health Management Information Systems (HMIS). However, there are limitations in the availability and quality of mortality data at national level in Viet Nam. This study assessed the completeness of recorded deaths and the reliability of recorded causes of death (COD) in the A6 death registers in the national routine HMIS in Viet Nam.

**Methodology and findings:**

1477 identified deaths in 2014 were reviewed in two provinces. A capture-recapture method was applied to assess the completeness of the A6 death registers. 1365 household verbal autopsy (VA) interviews were successfully conducted, and these were reviewed by physicians who assigned multiple and underlying cause of death (UCOD). These UCODs from VA were then compared with the CODs recorded in the A6 death registers, using kappa scores to assess the reliability of the A6 death register diagnoses. The overall completeness of the A6 death registers in the two provinces was 89.3% (95%CI: 87.8–90.8). No COD recorded in the A6 death registers demonstrated good reliability. There is very low reliability in recording of cardiovascular deaths (kappa for stroke = 0.47 and kappa for ischaemic heart diseases = 0.42) and diabetes (kappa = 0.33). The reporting of deaths due to road traffic accidents, HIV and some cancers are at a moderate level of reliability with kappa scores ranging between 0.57–0.69 (p<0.01). VA methods identify more specific COD than the A6 death registers, and also allow identification of multiple CODs.

**Conclusions:**

The study results suggest that data completeness in HMIS A6 death registers in the study sample of communes was relatively high (nearly 90%), but triangulation with death records from other sources would improve the completeness of this system. Further, there is an urgent need to enhance the reliability of COD recorded in the A6 death registers, for which VA methods could be effective. Focussed consultation among stakeholders is needed to develop a suitable mechanism and process for integrating VA methods into the national routine HMIS A6 death registers in Viet Nam.

## Introduction

Mortality statistics form a crucial component of national health information systems. Mortality indicators are essential for quantifying population health status and for measuring a country’s health development [1–3]. With a population of over 95 million [4], there is a critical need for such data for Viet Nam. However, there are limitations in the availability and quality of national mortality data in Viet Nam, in particular relating to the capture of causes of death (COD) data [3, 5–7].

Currently, information on mortality by sex and age are only derived from the annual mortality surveys conducted by the Government Statistics Office (GSO), and the limitations of these data are well known [8, 9]. In addition, there are three demographic surveillance sites which record mortality data by age, sex and cause, but these are limited in term of sample size as well as representativeness at the national level [10–13]. A national sample mortality surveillance project across a representative sample of 192 communes in Viet Nam was conducted as a special study to collect data on deaths which occurred during 2008–2009 [3]. The data from this activity yielded the first ever nationally representative mortality data by age, sex, and causes of death. However, this surveillance-based data collection ended at the conclusion of the project period, and there has been no follow up since then. Hence, the absence of regular and reliable national mortality data remains a critical issue in Viet Nam.

There are two main systems for collection and compilation of primary mortality data, which could serve as the basis for routine national mortality statistics: the national Civil Registration and Vital Statistics system (CRVS); and the national routine Health Management Information System (HMIS). The national CRVS is operated by the Ministry of Justice (MOJ) across the country. In each commune, the office of the justice clerk maintains a civil and vital events register to record births, deaths, and marriages in the resident population, which are then reported to district, provincial and central levels. However, the registration of deaths recorded in this system is incomplete. The information on the COD is often unavailable or poorly recorded [5, 14, 15] since it depends totally on whether and how the family report information about the death of their relatives to the commune justice officers.

The HMIS is operated by the Ministry of Health (MOH) to routinely collect health information which serves the health sector’s planning and management purposes. The “A6 death register” is a log-book used to routinely record every death events which occur in each commune across the country–the smallest official administrative unit in Viet Nam [16]. *(More details about the Vietnamese administrative hierarchy is presented in S1 Fig)*. Accordingly, the local commune health staff at the commune health stations (CHS) identify and record basic demographic data and information on the COD for each death into the A6 death register [16]. However, the quality of the A6 death registers has not been comprehensively assessed. A few epidemiological studies have been conducted using data from the A6 death registers, but these studies have focussed on recorded causes of death for specific diseases or conditions only [17, 18]. They did not assess the quality of data from the A6 death registers in terms of the completeness of death registration, and the validity of recorded causes of death.

The HMIS’s A6 death register system could provide the most comprehensive mortality data for the health sector and could be the optimal national mortality data source, since it is designed to capture all deaths in the community. More importantly, the MOH’s A6 death registers could provide information on COD for each individual death, which is essential to address the current limitations in the national CRVS. In January 2017, the National Action Plan for CRVS for the period 2017–2024 submitted by MOJ was approved by the Vietnamese Prime Minister [19]. This action plan indicated the need for recording of information on COD on death certificates. The MOH has been assigned the responsibility of providing the COD and ensuring their quality.

A thorough assessment of the two major aspects of mortality data quality—the completeness of death reporting, and the accuracy of reported CODs—will help identify the key strengths and limitations of the A6 death register system. This study comprises an evaluation of these two aspects of data quality in the current HMIS A6 death register reporting system. Similar assessments of national mortality and cause of death reporting systems using primary data verification methods have been conducted in recent times in China [20, 21], Thailand [22] and Malaysia [23]. In general, these studies identified the need to revise structural and technical elements in the death reporting and cause of death ascertainment processes, and have led to interventions to strengthen their reporting systems.

It is anticipated that our study will provide the initial evidence for strengthening the MOH A6 death register system that will serve as a reliable source of national mortality statistics in Viet Nam.

## Materials and methods

### Study setting and design

The study was implemented in 2 provinces, Quang Ninh and Thai Nguyen, located in the Northern region of Vietnam. These provinces were chosen based on three criteria. First, these provinces participated in the previous sample mortality surveillance system [3]; therefore, this assessment benefited from the involvement of health staff experienced in the processes for collating mortality records from different sources as well as in using verbal autopsy methods. Second, these provinces have a close association with the Vietnamese institutions leading this research, namely the Hanoi Medical University (HMU), and the Hanoi University of Public Health (HUPH). A considerable number of staff in the Provincial Health Departments and District Health Centres are alumni of HMU and HUPH, and were more likely to be actively involved in the study. Third, since an allied objective of this study was related to evaluating mortality records from the HIV/AIDS surveillance program, we purposively chose these two provinces—Quang Ninh and Thai Nguyen, which are among the top 10 provinces with the highest HIV/AIDS burden in Vietnam in 2014 [24].

**Fig 1:**
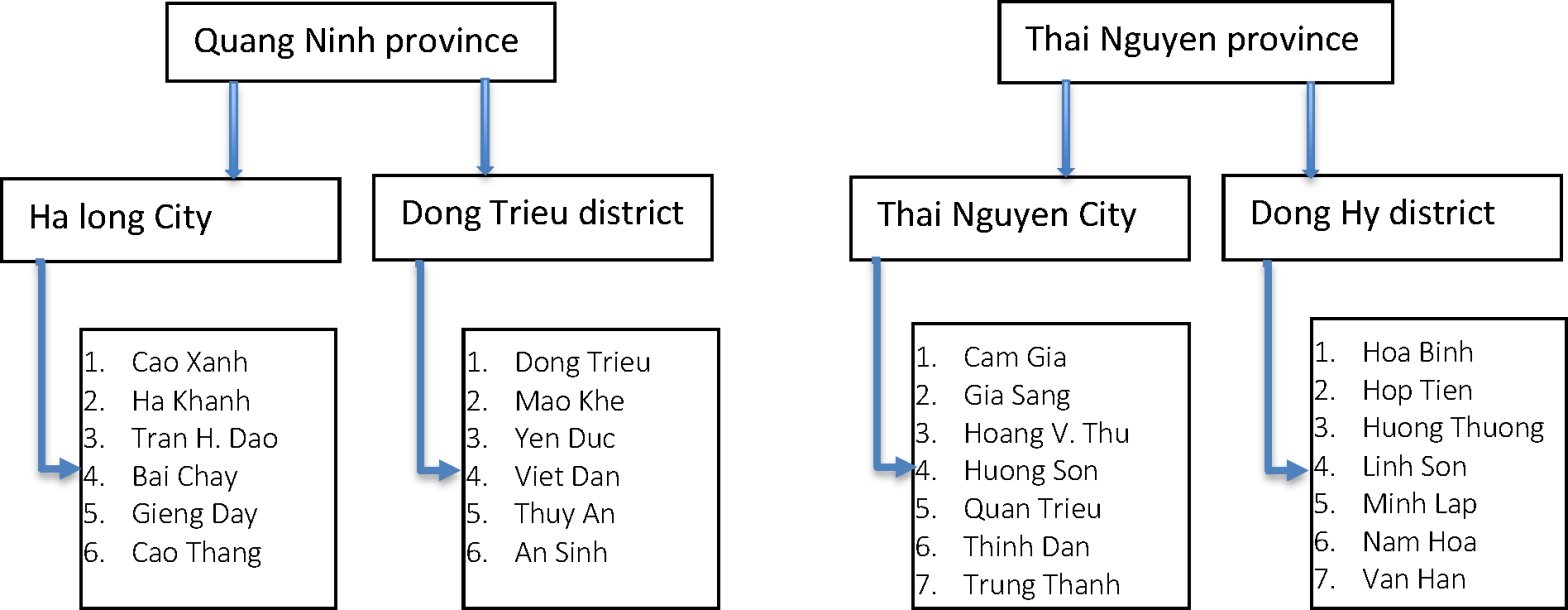
List of study sites in Quang Ninh and Thai Nguyen provinces.

In each province, one urban and one rural district were chosen to assess likely differences between urban and rural areas. These districts were selected based on prior information on estimated numbers of cases of HIV/AIDS. Within each selected district, 6–7 communes with the highest number of HIV deaths were chosen as study sites based on the availability of data on HIV cases from the surveillance program [24]. The study sites are described in Fig 1.

A cross-sectional design was employed for this study. The study population comprised all identified deaths among residents of the sample communes that occurred between 01/01/2014 and 31/12/2014. Mortality records were sourced from the A6 death registers, the death registers maintained by the commune justice clerk, and several other local health surveillance programs. Inclusion of all deaths was necessary to assess the completeness of the A6 death register reporting system. For each death, relatives of the deceased served as respondents for the verbal autopsy interviews.

### Data collection methods

As preparation for the field work in the two provinces, the principal investigator obtained a letter of support from her institute to introduce the purpose and significance of the study, to seek agreement for the provision of data/information and ask for support from the CHS during two pre-data collection training workshop as well as during the data collection in the field. The letters of support were sent to the heads of the provincial health departments and the heads of the provincial statistics offices of the two study provinces to obtain their permission as well as support to conduct the study and use the available death lists.

As an initial step, a training workshop was held in each province for two data collectors from each of the study communes. The data collectors were health staff working at each CHS, whose work related to management of the A6 death registers. The training workshop covered the data collection tools and methods for the two main components of this study, which are the assessment of completeness of data recorded in the A6 death registers, and the conduct of Verbal Autopsy (VA) interviews and their subsequent analysis.

#### Creating the list of deaths in each commune

As a first step in the data collection process, the trained CHS staff matched death cases from the A6 death register with the deaths in the CRVS death register maintained by the justice officer in each commune. Variables used for the matching process were name, sex, age, date of death and address of the deceased. A range of 5 years was allowed for matching age and a range of one month for matching date of death. In some instances, the name of the deceased was verified during the fieldwork. Regarding the address, it should be noted that each commune comprises several villages and the exact recording of specific address may vary. Hence, if all other information was matched except the village where the deceased lived, it was considered as a “conditional” matched case, which meant that the correct address needed to be confirmed during the field data collection.

A process for quality control of the matching process was included in this stage. If there was uncertainty regarding the matching of cases, due to marginal variations in certain variables, consultations were undertaken with key local informants including the head of the village, other community leaders and the Justice Clerk among others, to verify whether the case matched or was unique. This helped avoid duplication in the recording of death events.

After this matching procedure, the lists of deaths from different sources were merged to make a “combined list”, which reflected the unique set of deaths that occurred in the selected areas during the study period. Finally, the merged combined list was compared with lists of deaths available from other data sources at commune level, such as the Maternal and Child Health program and the district HIV/AIDS surveillance program, and updated to include any missed events, to develop the final list of identified deaths in the study sample communes. This final combined list included both matched cases, and those unique un-matched cases found in at least one of the available sources.

#### Verbal autopsy interviews

Questionnaire-based interviews were administered to the principal caregiver for the deceased in each household for each individual death on the final combined list. The interviewer explained the purpose of the assessment to the identified respondent to obtain their consent and collaboration. The respondent was asked to sign a consent form. The interviews were conducted only for consenting respondents. The interviewers were local medical personnel at the commune where the death occurred. Data collectors demonstrated strong communication and persuasion skills and were supported by principal investigators and supervisors, who were staff of the District Health Centres who were responsible for monitoring and providing assistance to data collectors.

The VA questionnaire used for this assessment was the updated version of the Vietnamese verbal autopsy questionnaire from the previous project [3, 25], which had been adapted from the WHO VA questionnaire.

#### Diagnosis and coding of underlying causes of death

All completed verbal autopsy questionnaire were sent to Hanoi Medical University for processing by a working team of three physicians led by one of the authors (NPH), who had previously undertaken an international training course on mortality coding in Australia. The other two physicians had been previously trained in cause of death diagnosis from VA questionnaires, and had participated in a previous mortality surveillance project [3]. In this study, each completed VA questionnaire was reviewed by a single physician, who identified causes of death for each case (including direct, antecedent, underlying and contributory causes) based on available evidence from the verbal autopsy form. Using this information they filled in an international death certificate for each case. If there was any uncertainty in assigning causes of death in an individual case, the form was reviewed and discussed by the team to achieve consensus, under the guidance of the team leader, who supervised and supported the other two physicians, and where necessary, adjudicated on the final decision on the causes of death for the case under discussion.

In the next step, all causes recorded in the death certificate were assigned an ICD-10 code [26] and subsequently international mortality coding rules were applied to identify and code the underlying cause for each death [27]. For all 1365 cases with completed VA interviews, this task was conducted by the co-author (NPH) who had undertaken the international training course on mortality coding.

The causes of death from the A6 death registers also needed to be assigned ICD-10 codes for subsequent data analysis. This task was performed by the first author of this manuscript (TTH) who had also been trained in mortality coding previously and during her graduate studies.

Finally, the Underlying Causes of Death (UCOD) which were derived from the A6 death registers as well as from the VA reinvestigation were then aggregated using the WHO ICD-10 mortality tabulation list 1 comprising 103 cause categories, for descriptive analyses [28].

**Fig 2:**
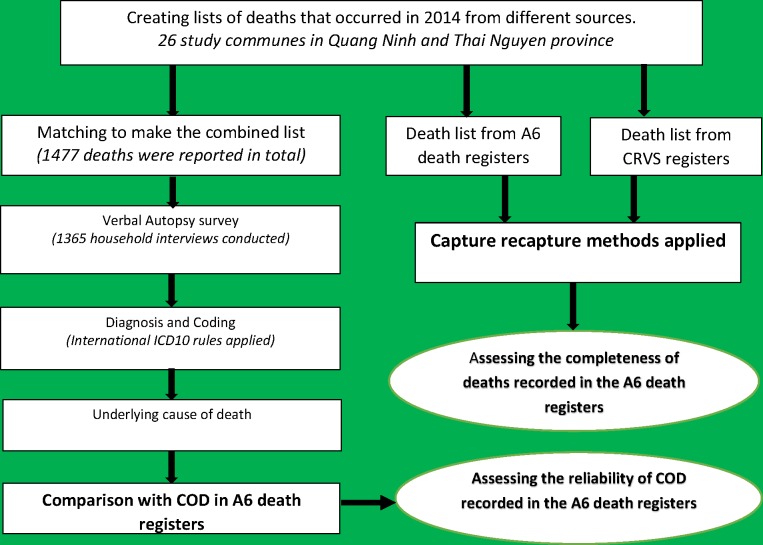
Process for assessment of completeness of deaths recorded and reliability of COD in the A6 death registers.

The study process is described in Fig 2.

### Statistical analysis

The measure of completeness of deaths reported in the A6 death registers was assessed by applying the capture-recapture method [29] to the two independent sources (the A6 death registers and CRVS death registers at commune level). The completeness of each data source was calculated as the proportion of the number of deaths recorded in it out of the total estimated deaths derived from the capture-recapture method. Proportions were calculated and compared across communes and urban/rural areas. Standard errors and 95% confidence intervals were computed for each proportion [3, 30].

For the assessment of reliability of causes of death recorded in the A6 death registers, kappa statistics were estimated to test the level of agreement [30–32] between the A6 death registers and the results of the VA survey for selected leading CODs. Kappa scores from 0 to under 0.4 suggests low reliability; kappa from 0.4 to under 0.7 means moderate reliability; and kappa over 0.7 means good reliability [33].

Also, a descriptive matrix of distributions of agreement and misclassification by cause across the two data sources was constructed [34]. Further, computation of the Sensitivity and Positive Predictive Value (PPV) for selected leading causes of death was carried out considering VA as the reference diagnosis.

### Ethical considerations

Ethical approval for this study was obtained from the Institutional Review Board of Ha Noi Medical University, Viet Nam (granted number 173/HMU-IRB) and the University of Queensland, Australia (granted number THT23062015). All respondents were provided with an explanation about the purpose of the study and were asked to sign an informed consent form before the interviews took place. Respondents could refuse to participate in, or withdraw from the interview at any time.

## Results

### General information about the study population

There were 15 urban and 11 rural communes chosen for this study. Although the sample was stratified across urban and rural communes in both provinces, there was a higher representation of the urban population in the study sample as compared to the national urban proportion. This was because an allied objective of the study was related to deaths from HIV/AIDS, the prevalence of which is known to be higher in the urban areas of these provinces.

A total of 1477 deaths were recorded in the reference year, which comprised 746 cases in Quang Ninh province and 731 cases in Thai Nguyen province. Out of these 1477 deaths, the COD were re-investigated in 1365 cases using VA household interviews, equivalent to 92.6% of the total number of deaths. A VA interview could not be carried out in 112 cases (7.4% of total deaths), with losses to follow up mainly due to the migration of residents after the death of their family member.

Table 1 describes the key characteristics of the sample of deaths identified during the study. A considerable gender differential in numbers of deaths recorded was observed in both provinces. In regard to age, over two thirds of the deaths were among the elderly (60+). The number of deaths recorded under the age of 5 years is very low (24 cases).

**Table 1:** Descriptive statistics of study sample.

Study characteristic	Quang Ninh	Thai Nguyen	Total
Urban population/ commune	141,427 (8)	84,898 (7)	226,325 (15)
Rural population/ commune	20,199 (4)	52,173 (7)	72,912 (11)
Total deaths in the combined list	746	731	1,477
			
Total deaths by VA survey	670	695	1,365*
SEX: Male/Female	401/269 (60%/40%)	434/261 (62.4%/37.6%)	835/530 (61.2%/38.8%)
AGE group			
0–4	9 (1.3%)	15 (2.2%)	24 (1.8%)
5–14	8 (1.2%)	9 (1.3%)	17 (1.2%)
15–59	218 (32.5%)	237 (34.1%)	455 (33.3%)
60+	435 (64.9%)	434 (62.4%)	869 (63.7%)
Place of death (n = 1365)			
Home	596 (89.0%)	605 (87.1%)	1201 (88.0%)
Health facility	30 (4.5%)	36 (5.2%)	66 (4.8%)
Other places	44 (5.7%)	54 (7.7%)	98 (7.2%)
Last treatment at heath facility (n = 1365)			
Yes	467 (69.7%)	492 (70.8%)	959 (70.3%)
No	177 (26.4%)	194 (27.9%)	371 (27.2%)
Don’t know/Unsure	26 (3.9%)	9 (1.3%)	35 (2.6%)
Type of health facility** in the last treatment (n = 959)			
Central/provincial hospitals	362 (78.4%)	408 (83.1%)	770 (80.8%)
District hospitals	158 (34.1%)	118 (24.1%)	276 (28.9%)
Commune health station	13 (2.8%)	32 (6.5%)	45 (4.7%)
Healers (traditional medicine)	7 (1.5%)	13 (2.7%)	20 (2.1%)

(*) Information of 1356 deaths is available in supporting document S1 File

(**) The patients may have visited more than one health facility

Only 4.8% of deaths occurred at a health facility (including hospitals, commune health stations, clinics, etc.), however, in about 70% of events, the deceased had attended a health facility for their last treatment prior to death. Most of them had visited Central/Provincial Hospitals (80%) and in about 30% of cases, a visit to a district hospital was reported. Further analysis revealed that 400 out of 959 cases who attended hospitals had been discharged within 10 days prior to death.

### Completeness of deaths recorded in the A6 death registers

Table 2 describes the completeness of deaths recorded in the A6 death registers, which was measured by the Chandrasekar-Deming Capture-Recapture method, using the two independent sources of death data: the A6 death registers and CRVS death registers maintained at commune level. The completeness of the A6 death registers was 89.3% (95% CI 87.7–90.8). The completeness of the A6 death registers ranged from 72% to 100% in different communes in Quang Ninh and Thai Nguyen provinces (S1 Table). The results showed some differences in completeness in the A6 death registers between rural and urban areas in the two provinces, but these were not statistically significant.

**Table 2:** Completeness analysis*.

Commune	Deaths recorded in the A6 register	Deaths recorded in CRVS register	Matched cases between A6 and CRVS	Estimated number of deaths missing from A6 and CRVS	All Deaths (estimated by Capture Recapture method)	Completeness of A6 by capture-recapture method
Total	1,335	1,231	1,099	28	1,495	89.3% (87.7;90.8)
QUANG NINH province	661	637	558	15	755	87.6% (82.5;89.9)
Urban	554	525	456	14.8	638	86.9% (84.2;89.5)
Rural	107	112	102	0.5	117.5	91.1% (85.9;96.2)
THAI NGUYEN province	674	594	541	13	740.0	91.1% (89;93.1)
Urban	402	359	333	5.4	433.4	92.8% (90.3;95.2)
Rural	272	235	208	8.3	307.3	88.5% (84.9;92.1)

*A detailed table on the completeness by commune is presented in supporting document S1 Table.

### Reliability of causes of death recorded in the A6 death registers

Table 3 demonstrates the reliability of COD recorded in the A6 death registers, in comparison with the results from the VA. These results indicate that no COD were recorded with good reliability in the A6 death registers. HIV and some site-specific cancers (lung cancer, liver cancer, stomach cancer, and mouth and oropharynx cancers) demonstrated moderate levels of reliability with kappa scores ranging between 0.57–0.69 (p<0.001). Major cardiovascular conditions, such as stroke and ischaemic heart disease, also only demonstrated moderate levels of reliability, while there was very low reliability in the recording of diabetes (kappa = 0.33). Surprisingly, the A6 death register demonstrated only a moderate level of reliability (kappa = 0.69) for road traffic accident deaths, despite this being a reasonably straightforward COD.

**Table 3:** Reliability of CODs recorded in the A6 death registers by 12 leading causes.

Rank	Cause of death	ICD10 code	No of cases in A6	No of cases in VA	No of common cases	Kappa (95% CI) (p<0.001)	Sensitivity* (95% CI)	PPV* (95% CI)
1	Stroke	I60-I69	361	281	189	0.47 (0.41;0.52)	67.3 (61.8; 72.8)	52.4 (47.2; 55.6)
2	Lung cancer	C43	79	113	59	0.59 (0.50;0.67)	52.2 (43.0; 61.4)	74.7 (65.1; 84.3)
3	Liver cancer	C22	61	64	38	0.59 (0.49;0.69)	59.4 (47.4; 71.4)	62.3 (50.1; 74.5)
4	Pneumonia	J18	21	54	5	0.11 (0.01;0.22)	9.3 (1.6; 17.0)	23.8 (5.6; 42.0)
5	Road traffic accident	V01-V04; V06, V09-V80; V87, V89, V99	31	50	28	0.69 (0.56;0.80)	56 (42.2; 69.8)	90.3 (79.9; 100)
6	HIV/AIDS	B20-B24	30	38	22	0.64 (0.50; 0.77)	57.9 (42.2; 73.6)	73.3 (57.5; 89.1)
7	Cirrhosis of liver	K70, K74	35	38	18	0.48 (0.34; 0.62)	47.4 (31.5; 63.3)	51.4 (34.8; 68.0)
8	Ischaemic heart disease	I20-I25	22	35	12	0.42 (0.25; 0.57)	34.3 (18.6; 50.0)	54.5 (33.7; 75.3)
9	Other cardiovascular diseases	I00-I09; I26-I51; I70-I99	75	34	16	0.27 (0.15; 0.38)	47.1 (30.3; 63.9)	21.3 (12.0; 30.6)
10	Stomach cancer	C16	31	34	20	0.61 (0.46; 0.75)	58.8 (42.3; 75.3)	64.5 (47.7; 81.3)
11	Diabetes mellitus	E10-E14	17	31	8	0.33 (0.15; 0.49)	25.8 (10.4; 41.2)	47.1 (23.4; 70.8)
12	Mouth and oropharynx cancers	C01-C14	29	26	17	0.61 (0.46; 0.76)	65.4 (47.1; 83.7)	58.6 (40.7; 76.5)

* The VA diagnosis was considered the reference standard for evaluating sensitivity and positive predictive value

In this table, Sensitivity and Positive Predictive Values were calculated, considering the VA diagnosis as the reference standard. While the sensitivity scores largely reflect the levels of accuracy as inferred from the kappa statistic for each cause, the results showed that for several important causes including lung cancer, HIV/AIDS, and road traffic accidents, the A6 death registers showed reasonably high positive predictive values, indicating that in cases for which these diagnoses were recorded in the A6 death register, there was a greater likelihood of the diagnosis being confirmed by the VA. Nevertheless, there were considerable numbers of deaths reclassified to these causes from other categories (54 lung cancer deaths, 16 HIV/AIDS deaths, and 22 road traffic accident deaths), which resulted in the overall lower levels of reliability and sensitivity of the A6 death registers.

In order to further understand the differences in assigning COD in the A6 death register and in the VA, a misclassification matrix was created for 12 leading CODs as presented in Table 4. The rows of the table present the number of deaths recorded in the A6 while the columns present the number of deaths diagnosed by VA. As can be seen, there was considerable misclassification within the cardiovascular diseases group. For example, there were 361 cases recorded in A6 death registers as stroke deaths but only 189 cases were confirmed by VA. The remainder of the cases (172 deaths) were assigned to other causes such as ischaemic heart diseases, pneumonia, diabetes or other cardiovascular diseases by VA. Conversely, ischaemic heart diseases and other cardiovascular diseases recorded in the A6 death registers could be re-diagnosed as stroke by VA.

**Table 4:** Misclassification of the diagnoses between the A6 death registers and verbal autopsy.

		Verbal Autopsy														
A6 death register	Leading causes of death (L)	L1	L2	L3	L4	L5	L6	L7	L8	L9	L10	L11	L12	Other diseases	Ill-defined COD	Total
1	Stroke (L1)	**189**	9	7	14	6	2	4	7	2	3	6	3	52	57	**361**
2	Lung cancer (L2)	5	**59**	2	1			1		1	3	1		6		**79**
3	Liver cancer (L3)		4	**38**	1			2	2			1	1	12		**61**
4	Pneumonia (L4)	3	1		**5**		1						1	7	3	**21**
5	Road traffic injury (L5)	1				**28**						1		1		**31**
6	HIV/AIDS (L6)		1		2	1	**22**							4		**30**
7	Cirrhosis of liver (L7)	2	1	2	1		1	**18**				2	1	5	2	**35**
8	Ischaemic heart disease (L8)	4		1					**12**	1				1	3	**22**
9	Other CVD (L9)	8			7				5	**16**		2		21	16	**75**
10	Stomach cancer (L10)		3	2							**20**	1		5		**31**
11	Diabetes mellitus (L11)	2	1		1	1						**8**		4		**17**
12	Mouth and oropharynx cancers (L12)		2							1	1		**17**	8		**29**
	Other diseases	18	9	4	9	13	3	4	3	3	2	2	1	**190**	21	**282**
	Ill-defined and unknown COD	49	23	8	13	1	9	9	6	10	5	7	2	59	**90**	**291 (21.3%)**
** **	**Total**	**281**	**113**	**64**	**54**	**50**	**38**	**38**	**35**	**34**	**34**	**31**	**26**	**375**	**192 (14%)**	**1365**

Table 4 identifies the significant contribution made by the VA in classifying deaths through the assignment of ill-defined causes in the A6 death register to more specific causes. As can be seen, there were 291 ill-defined and unknown causes (21% of the deaths) recorded in A6 death registers, of which 201 cases were reclassified by the VA into other leading specific causes of death such as stroke, lung cancer, pneumonia, cirrhosis, ischaemic heart disease, among others.

## Discussion

Findings from this study are important as a primary step towards strengthening the mortality reporting system in Viet Nam. It provides empirical evidence of the current situation of mortality data recording in the national routine HMIS. The study also provides useful observations on the utility of VA methods to identify causes for deaths occurring outside health facilities in Viet Nam.

### Discussion regarding main findings

The completeness of deaths recorded in the A6 death registers in the study sites was relatively high overall (89%). This is a positive result since it confirms the value of the A6 death registers in counting and recording deaths that occurred in the study areas. This result shows better completion of the A6 death registers in comparison to the very early study conducted in Bavi district of Hanoi [35], and the sample surveillance project implemented in a representative sample of 192 communes in different regions of Viet Nam [3]. Quang Ninh and Thai Nguyen were provinces chosen in the earlier sample based mortality surveillance conducted during 2008–2009, and it is possible that the systems in these provinces would have benefited from those activities.

This study is one component of a larger research project designed to investigate the performance of the MOH A6 death register system and provide solutions to strengthen it. The quantitative research reported here was supplemented by a qualitative component (not described in this manuscript) that investigated the reasons for the problems with data quality, as well as potential solutions to strengthen the system. Although completeness of number of deaths recorded is high overall, it varies among different communes. During the qualitative enquiry, it was found that in several communes (particularly those with very high levels of completeness), the CHS staff consult with the CRVS death records. The qualitative enquiry clearly identified the value in routine local data sharing and triangulation as a solution to improve data completeness.

From another perspective, the small number of reported child deaths results in very low estimated under 5 mortality rates for the study population. Similarly implausible low child mortality rates were also observed in the previous national sample mortality surveillance system that operated in 2008–2009 [3]. More detailed investigation of the under-five mortality reporting is necessary to confirm if under-five mortality is truly low in the sample communes for this study, or if there is a bias from under-reporting of child deaths.

In contrast to the relatively high level of completeness, the reliability of different CODs recorded in the A6 death registers ranged from low to moderate, as per the scales defined in the Methods section. There are some explanations for this. First, information recorded in the A6 death registers is based on the response to the open-ended question enquiring about the COD posed by the CHS staff to the family member or someone in the commune, at the time of recording the event in the A6 death register. The response is usually very short and simple, and could be a specific disease/ injury, or some vague terms including “ill/sick”, “senility”, “unidentified cause”. In some instances, this column could even be left blank, because the respondent could be a distant relative or another member of the village who was unaware of the circumstances of the death. In general, the information is recorded in the A6 death registers without any clarification, and this affects its reliability.

Secondly, the A6 death register has only a single column for recording the cause of death, and there is no potential to record multiple causes. There is no instruction as to whether the reported cause is the underlying COD or the direct COD. The detailed VA interview enabled the identification of an underlying cause, resulting in reclassification in a number of instances. In cases in which there were multiple comorbid conditions, the commune heath staff documented only one cause while the VA allowed documentation of multiple CODs, following which ICD rules [36] were applied to select the correct underlying cause, which could have been a condition other than the one recorded in the A6.

Another factor that could influence our observations on the reliability of COD in the A6 death registers is the level of aggregation of causes used for the comparative analysis. We assessed the reliability and validity of COD for certain important specific causes (such as stroke, road traffic accident, stomach cancer and liver cancer). If we were to use a higher level of aggregation such as at the level of ICD-10 Chapters (for example “diseases of the circulatory system” or “neoplasms”) or a broader categorization (such as Communicable diseases, Non-communicable diseases, Injuries, and Other), our measurement of reliability and validity would be considerably different from those for the more specific causes. For example, based on the data from the current study, the Sensitivity and PPV of non-communicable diseases were 80% and 82% respectively, which were higher than those of the more specific such as stroke (67.3% and 52.4%), or liver cancer (59.4% and 62.3%). Similarly, Sensitivity and PPV of Injury were 72% and 91% respectively, which was higher than road traffic accidents (56% and 90% respectively). The level of aggregation of causes used in the comparative analysis therefore is very important, since it effects the interpretation of the study results. A study conducted in three provinces of Viet Nam by Stevenson et al. in 2008 indicated that sensitivity and PPV of injury deaths recorded in A6 death registers were 75% and 88% respectively, which were considered to represent good validity. These authors concluded that the A6 death registers system performed well in relation to the completeness and classification of injury related deaths [37]. However, this statement needs to be considered carefully when looking at the validity of the A6 death registers in recording specific types of external causes that lead to injury such as road traffic accidents, falls and drowning. For intervention purposes, it is necessary to have as specific information as possible. In this regard, the conclusions from our study were based on the results of the data analysis for more specific causes of death.

This study found considerable misclassification in the diagnosis of stroke as the UCOD between the A6 death registers and VA. This phenomenon can be explained in different ways. In Viet Nam, the term “stroke” seems to be a very common term used in the community when people talk about a death that occurred suddenly in a person with a history of hypertension or cardiovascular disease. Community members had a different understanding of “stroke” which might have affected the diagnosis of stroke in some aspects [38]. In the Vietnamese language, “strokes” are called “tai biến mạch máu não” (cerebrovascular accident) or “đột quỵ” (stroke) or “đột tử” (instantaneous death). Also, in some cases, “stroke” may be used for people who died due to an unknown cause (for example, a person who died during their sleep). The family may assume that the death was due to a stroke, reporting this as the cause of death to commune health workers, who then recorded it as such in the A6 death register. These situations may explain the low agreement between the diagnoses of stroke in the A6 death registers and the VA based causes of death.

### Suggestions for improving data quality in the A6 death registers

About 11% of actual deaths were not captured by the national HMIS A6 death registers. The completeness of the registers could be improved by routinely matching them with the CRVS death registers, as well as reviewing different local data sources such as Maternal and Child health books or/and records of the HIV surveillance program, among others. In particular, there is a need for a concerted effort to improve the identification of stillbirths, and neonatal and infant deaths, for more reliable measurement of early age mortality for small areas in Viet Nam [39, 40].

VA methods have been widely used in many countries in the world [41, 42], in different settings and diseases specific areas [43–47]. This study confirms the usefulness of VA methods in identifying the COD as well as re-classifying the conditions reported as ill-defined in the A6 death registers at CHSs in Viet Nam setting. The A6 death registers can only provide one COD for each individual death and there is no indication whether it was underlying COD or not. The detailed VA interview, however, enabled the collection of information on signs and symptoms of the deceased as well as other information relating to the patients’ medical histories, which helped to produce a more accurate COD. VA allows the collection of multiple CODs which is useful to understand more about other contributing causes that are not the underlying COD but contribute to the death, especially in elderly people and people suffering from certain specific diseases or disability [48]. This facilitates a better sense of the burden of disease in a community.

In Viet Nam, VA methods have been used in Health and Demographic Surveillance Surveys and a number of projects/studies to identify cause-specific death rates and to measure the burden of diseases [10, 11, 49]. These studies has been conducted in both rural and urban areas of Viet Nam [3, 10–12, 50, 51]. Therefore, utilizing the advantage of VA methods for routinely mortality data collection at CHS could help to provide more reliable COD for the national HMIS A6 death registers in Viet Nam.

Our study found about 70% of the deceased were hospitalized for their last treatment and 42% of them died within 10 days after discharge. This finding suggests that a good system of discharge documentation may help to improve the availability of reliable information to ascertain the causes of death from the verbal autopsy. A recent study on assessing the quality of evidence for verbal autopsy diagnosis of stroke in Viet Nam also found that respondents of cases with recent hospital admissions or visits have a better recall of disease symptoms than those without hospital admission [38]. Therefore, the issuing of a hospital discharge summary for every patient and an appropriate mechanism of information sharing between hospitals and CHS would be useful for supporting the improved performance of VA data collection at CHS in Vietnam.

### Strengths and limitations of the study

There are number of strengths of the study. Firstly, it is one of very few studies assessing the completeness and reliability of the HMIS A6 death register reporting system in Viet Nam for all CODs. The measurement of reliability was conducted for specific CODs, providing better understanding of the strengths and limitations of this recording system. Thirdly, the tools and guidelines had been carefully translated into Vietnamese, used in an earlier project [3], and had been iteratively adjusted based on field experience during that project. Additionally, quality controls were applied strictly at each of the project stages to ensure data quality for this study.

Despite these strengths, this study has some limitations. First, the sample size is relatively small which limited the analysis by sex and age groups. There appears to be underreporting of stillbirths and neonatal and infant deaths which limits interpretation of the findings for the youngest age groups. Second, the issue of recall bias also needs to be considered. This study focused on deaths that occurred in 2014 but data collection was carried out from May to July 2015, which resulted in recall periods ranging from 5 months to 17 months. The literature shows that most previously reported VA studies had a maximum 12 months recall period [33].

## Conclusion

The study identified that the completeness of deaths recorded in the A6 death registers by commune health stations is relatively high, but still needs to be improved by examining CRVS death registers and other local data sources. The reliability of COD recorded in the A6 death register is low to moderate, and adding VA methods would be effective in enhancing quality. We recommend that the MOH develop a suitable mechanism and process to apply VA methods in combination with COD data collection at CHS in the national routine HMIS in Viet Nam. Further studies on VA validation and operation will guide those implementation activities.

## Supporting information

S1 Fig The administrative hierarchy in Viet Nam. (PDF)

S1 Table Completeness of deaths recorded in the A6 death registers by communes. (PDF)

S1 File Data for cases which were re-investigated causes of death. (SAV)
