# Supplementary material for: Completeness and reliability of mortality data in Viet Nam: Implications for the national routine health management information system
Source: PLoS One. 2018 Jan 25;13(1):e0190755. doi: 10.1371/journal.pone.0190755 (PMC5784908; doi:10.1371/journal.pone.0190755)
Supplement: S1 Fig — (PDF) [file pone.0190755.s001.pdf]

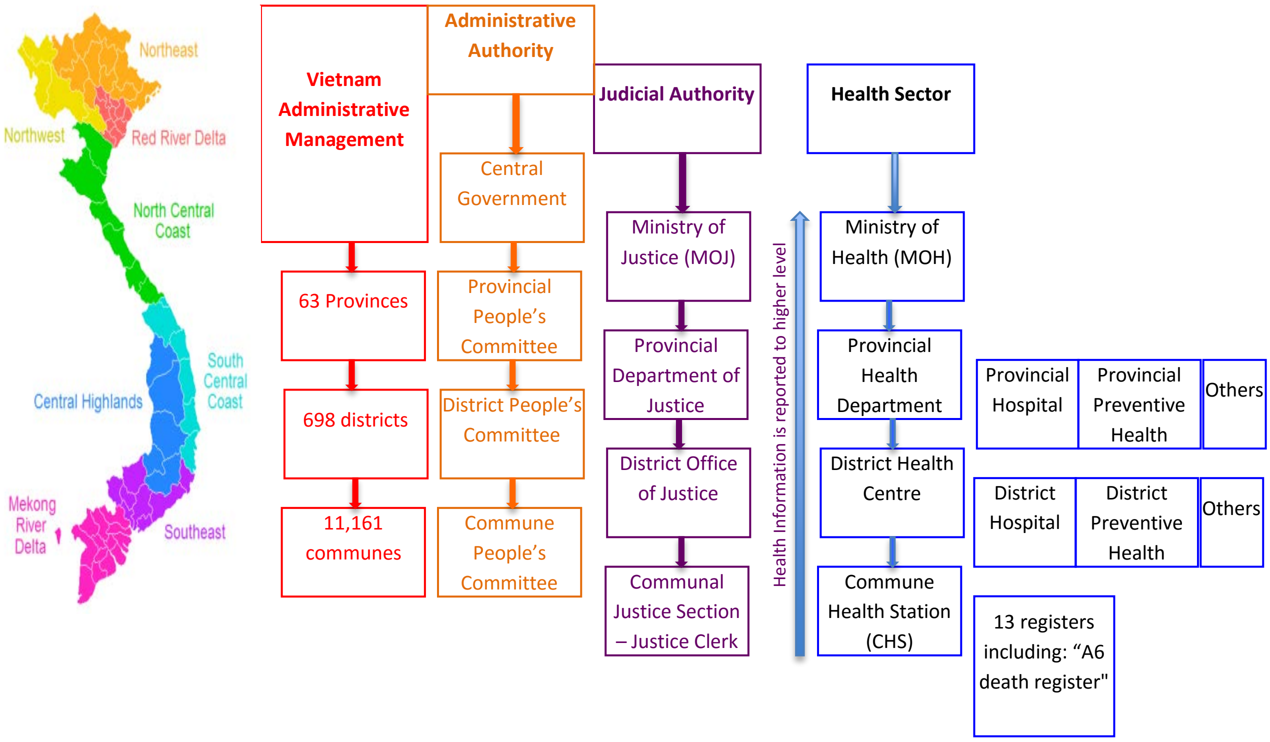

S1 Fig. The administrative hierarchy in Vietnam

Viet Nam encompasses 63 provinces, which are divided into 698 districts and those districts are divided into 11,161 communes (Vietnam General Statistics Office, 2014). In each province, the Provincial People's Committee takes the lead role in the overall administrative and development activities within the province. The Provincial Health Department and Provincial Department of Justice are under the management of Provincial People's Committee (depicted in S1. Fig by placing these boxes lower than the box for the Provincial People's Committee)
