# Supplementary material for: Completeness and reliability of mortality data in Viet Nam: Implications for the national routine health management information system
Source: PLoS One. 2018 Jan 25;13(1):e0190755. doi: 10.1371/journal.pone.0190755 (PMC5784908; doi:10.1371/journal.pone.0190755)
Supplement: S1 Table — (PDF) [file pone.0190755.s002.pdf]

S1 Table. Completeness of deaths recorded in the A6 death registers by communes

	Commnue	Deaths recorded in A6 death register	Deaths recorded in CRVS books	Matched cases between A6 and CRVS books	Proportion of matched cases in A6 death register	Deaths recorded in Other sources only	Estimated number of deaths missing from A6 and CRVS books	All Deaths estimated by Capture Recapture method	Deaths obtained (combined list)	Completeness of A6 by capture-recapture method	95% confident interval
	**Total**	**1,335**	**1,231**	**1,099**	**82.3%**	**10**	**28.3**	**1,495**	**1,477.0**	**89.3%**	**(87.7-90.8)**
**QUANG NINH province**		**661**	**637**	**558**	**84.4%**	**6**	**14.6**	**754.6**	**746.0**	**87.6%**	**(85.2-89.9)**
	**HA LONG CITY**	**343**	**360**	**304**	**88.6%**	**5**	**7.2**	**406.2**	**404.0**	**84.4%**	**(80.9-88.0)**
1	Cao Xanh	71	86	62	**87.3%**	0	**3.5**	**98.5**	**95.0**	72.1%	(63.2-81.0)
2	Ha Khanh	41	38	33	**80.5%**	0	**1.2**	**47.2**	**46.0**	86.8%	(77.2-96.5)
3	Tran Hung Dao	51	57	44	**86.3%**	0	**2.1**	**66.1**	**64.0**	77.2%	(67.1-87.3)
4	Bai Chay	51	53	49	**96.1%**	0	**0.2**	**55.2**	**55.0**	92.5%	(85.5-99.4)
5	Gieng Day	58	58	57	**98.3%**	5	**0.0**	**59.0**	**64.0**	98.3%	(95.0-100.0)
6	Cao Thang	71	68	59	**83.1%**	0	**1.8**	**81.8**	**80.0**	86.8%	(79.4-94.1)
	**DONG TRIEU district**	**318**	**277**	**254**	**79.9%**	**1**	**5.8**	**346.8**	**342.0**	**91.7%**	**(88.8-94.6)**
7	Dong Trieu	34	31	25	**73.5%**	0	**2.2**	**42.2**	**40.0**	80.6%	(68.7-92.6)
8	Mao Khe	177	134	127	**71.8%**	0	**2.8**	**186.8**	**184.0**	94.8%	(91.6-98.0)
9	Yen Duc	41	41	41	**100.0%**	1	**0.0**	**41.0**	**42.0**	100.0%	(100.0-100.0)
10	Viet Dan	18	21	17	**94.4%**	0	**0.2**	**22.2**	**22.0**	81.0%	(64.6-97.3)
11	Thuy An	24	26	20	**83.3%**	0	**1.2**	**31.2**	**30.0**	76.9%	(62.1-91.7)
12	An Sinh	24	24	24	**100.0%**	0	**0.0**	**24.0**	**24.0**	100.0%	(100.0-100.0)
**THAI NGUYEN province**		**674**	**594**	**541**	**80.3%**	**4**	**13.0**	**740.0**	**731.0**	**91.1%**	**(89.0-93.1)**
	**THAI NGUYEN CITY**	**402**	**359**	**333**	**82.8%**	**3**	**5.4**	**433.4**	**431.0**	**92.8%**	**(90.3-95.2)**
13	Cam Gia	68	58	55	**80.9%**	2	**0.7**	**71.7**	**73.0**	94.8%	(89.7-100.0)
14	Gia Sang	51	41	35	**68.6%**	0	**2.7**	**59.7**	**57.0**	85.4%	(76.4-94.3)
15	Hoang V Thu	62	56	54	**87.1%**	0	**0.3**	**64.3**	**64.0**	96.4%	(91.9-101.0)
16	Huong Son	49	42	38	**77.6%**	1	**1.2**	**54.2**	**54.0**	90.5%	(82.7-98.3)
17	Quan Trieu	61	58	58	**95.1%**	0	**0.0**	**61.0**	**61.0**	100.0%	(100.0-100.0)
18	Thinh Dan	51	48	37	**72.5%**	0	**4.2**	**66.2**	**62.0**	77.1%	(67.0-87.2)
19	Trung Thanh	60	56	56	**93.3%**	0	**0.0**	**60.0**	**60.0**	100.0%	(100.0-100.0)
	**DONG HY district**	**272**	**235**	**208**	**76.5%**	**1**	**8.3**	**307.3**	**300.0**	**88.5%**	**(84.9-92.1)**
20	Hoa Binh	19	19	19	**100.0%**	0	**0.0**	**19.0**	**19.0**	100.0%	(100.0-100.0)
21	Hop Tien	31	30	30	**96.8%**	0	**0.0**	**31.0**	**31.0**	100.0%	(100.0-100.0)
22	Huong Thuong	41	28	28	**68.3%**	0	**0.0**	**41.0**	**41.0**	100.0%	(100.0-100.0)
23	Linh Son	44	44	44	**100.0%**	1	**0.0**	**44.0**	**45.0**	100.0%	(100.0-100.0)
24	Minh Lap	40	35	25	**62.5%**	0	**6.0**	**56.0**	**50.0**	71.4%	(59.6-83.3)

25	Nam Hoa	45	36	29	**64.4%**	0	**3.9**	**55.9**	**52.0**	80.6%	(70.2-90.9)
26	Van Han	52	43	33	**63.5%**	0	**5.8**	**67.8**	**62.0**	76.7%	(66.7-86.8)
